# Association of extreme precipitation with hospitalizations for acute myocardial infarction in Beijing, China: A time-series study

**DOI:** 10.3389/fpubh.2022.1024816

**Published:** 2022-09-27

**Authors:** Yuxiong Chen, Zhen'ge Chang, Yakun Zhao, Yanbo Liu, Jia Fu, Yijie Liu, Xiaole Liu, Dehui Kong, Yitao Han, Siqi Tang, Zhongjie Fan

**Affiliations:** Department of Cardiology, Peking Union Medical College Hospital, Peking Union Medical College and Chinese Academy of Medical Sciences, Beijing, China

**Keywords:** extreme precipitation, acute myocardial infarction, distributed lag model, time-series analysis, hospitalizations

## Abstract

**Background:**

In the context of global climate changes, increasing extreme weather events have aroused great public concern. Limited evidence has focused on the association between extreme precipitation and hospitalizations for acute myocardial infarction (AMI). Our study aimed to examine the effect of extreme precipitation on AMI hospitalizations.

**Methods:**

Daily AMI hospitalizations, weather variables and air pollution data in Beijing from 2013 to 2018 were obtained. We used a time-series analysis with a distributed lag model to evaluate the association of extreme precipitation (≥95th percentile of daily precipitation) with AMI hospitalizations. Subgroup analysis was conducted to identify the vulnerable subpopulations and further assessed the attributable burden.

**Results:**

Extreme precipitation increased the risk of AMI hospitalizations with significant single-day effects from Lag 4 to Lag 11, and the maximum cumulative effects at Lag 0–14 (CRR = 1.177, 95% CI: 1.045, 1.326). Older people (≥65 years) and females were more vulnerable to extreme precipitation. The attributable fraction and numbers of extreme precipitation on AMI hospitalizations were 0.68% (95% CI: 0.20%, 1.12%) and 854 (95% CI: 244, 1,395), respectively.

**Conclusion:**

Extreme precipitation is correlated with a higher risk of AMI hospitalizations. The elderly (≥65 years) and females are more susceptible to AMI triggered by extreme precipitation.

## Introduction

Ischemic heart disease (IHD) remains the leading contributor to mortality and disability worldwide and in China (1, 2). It was estimated that there were 197 million prevalent cases of IHD in 2019, leading to 182 million disability-adjusted life years (DALYs) and 9.14 million deaths (1). In China, the DALYs of IHD increased by 125.3% from 1990 to 2017, ranking second place in all diseases (2). Acute myocardial infarction (AMI) is the most severe manifestation of IHD characterized by myocardial necrosis resulting in substantial morbidity and mortality worldwide (3, 4). Besides the well-known risk factors at individual levels, such as age, sex, smoking, hypertension, diabetes and dyslipidemia, several meteorological factors, such as temperature, air pressure and humidity, have been shown to play a role in the incidence of AMI (5–7).

Global climate shifts have caused an increasing number of extreme weather events, such as cold spells, heatwaves, and extreme precipitations (8–10). The adverse health effects of extreme weather conditions have aroused growing attention from researchers (11–13). Many studies have suggested that extreme precipitation is correlated with various diseases, especially infectious diseases (e.g., campylobacteriosis, dengue and hand-foot-mouth disease, etc.) (14–16), whereas the studies on extreme precipitation and cardiovascular diseases are limited. A recent study in Hefei, China has found a significant relationship between extreme precipitation and hospitalizations for ischemic stroke (17). Only a few previous studies have explored the associations between precipitation and AMI, and the results were inconsistent (5, 6, 18–25). For instance, two studies conducted in the United States and Switzerland, respectively showed positive correlations between precipitation and AMI hospitalizations in some specific regions (19, 20), whereas negative relationships were reported in two nationwide studies from Sweden and Japan, respectively (5, 25). However, no study has investigated the effect of extreme precipitation on AMI hospitalizations.

As one of the most populous cities in China, Beijing shoulders a huge burden of AMI, with an incident rate of 231.6 per 100,000 in 2016–2018 (26). Moreover, an upward tendency of extreme precipitation was observed in Beijing and China under the scenario of global warming (27, 28). The purposes of our study were to explore the association of extreme precipitation with AMI hospitalizations and to identify susceptible subgroups of the population in Beijing, China from 2013 to 2018. We further estimated the attributable burden of hospitalizations for AMI owing to extreme precipitation. The results may provide insights into the association between extreme precipitation and AMI occurrence, and assist to develop public health policies and preventive measures to reduce the adverse health effects due to extreme precipitation.

## Materials and methods

### Study location

Beijing is the capital of China with a resident population of 21.54 million in 2018 according to the Beijing Bureau of Statistics (http://nj.tjj.beijing.gov.cn/nj/main/2019-tjnj/zk/indexch.htm). It is located in northern China (39°26'N−41°03'N latitude, 115°25'E−117°30'E longitude), covering an area of 16,410.54 km^2^. Beijing belongs to a typical semihumid continental monsoon climate with four distinctive seasons including dry, cold winters and rainy, hot summers (29).

### Data collection

The data on daily AMI hospitalizations were obtained from all the public and private hospitals from the Beijing Municipal Health Commission Information Center, a citywide hospitalization surveillance system (http://www.phic.org.cn/) from 2013 to 2018. The admission records include the dates of admission and discharge, age, sex, residential address, and discharge diagnoses. The inclusion criteria were as follows: (1) patients with a principal discharge diagnosis of AMI based on the codes (I21-22) from the International Classification of Diseases, 10th Version (ICD-10); (2) patients residing in Beijing according to their residential addresses. We further excluded patients under 20 years old for a limited number of records (*N* = 21). The study was approved by the Institutional Review Board of Peking Union Medical College Hospital.

Daily weather data, including daily mean temperature (°C), relative humidity (%), barometric pressure (hPa), precipitation (mm) and sunshine duration (h) came from the China Meteorological Data Sharing Service System (http://data.cma.cn/). Daily air pollution data [fine particulate matter (PM_2.5_), inhalable particulate matter (PM_10_), sulfur dioxide (SO_2_), nitrogen dioxide (NO_2_), ozone (O_3_), and carbon monoxide (CO)] were retrieved from the China National Environmental Monitoring Center (http://www.cnemc.cn/).

### Study design and statistical analysis

So far, there is no standard definition of extreme precipitation. Because of the various climatic types and precipitation distribution characteristics in different geographic areas, extreme precipitation was usually defined based on the percentile method in previous studies, involving 90th, 95th and 99th percentile (16, 30, 31). Finally, we referred to several studies conducted in China (15, 17, 27, 32, 33), and divided precipitation into three ordinal categories taking the 95th percentile of the cumulative daily precipitation as the cut-off point: (1) no precipitation: equal to 0 mm (reference group); (2) moderate precipitation: more than 0 mm and less than 95th percentile; (3) extreme precipitation: more than or equal to 95th percentile (17, 32).

**Figure 1:**
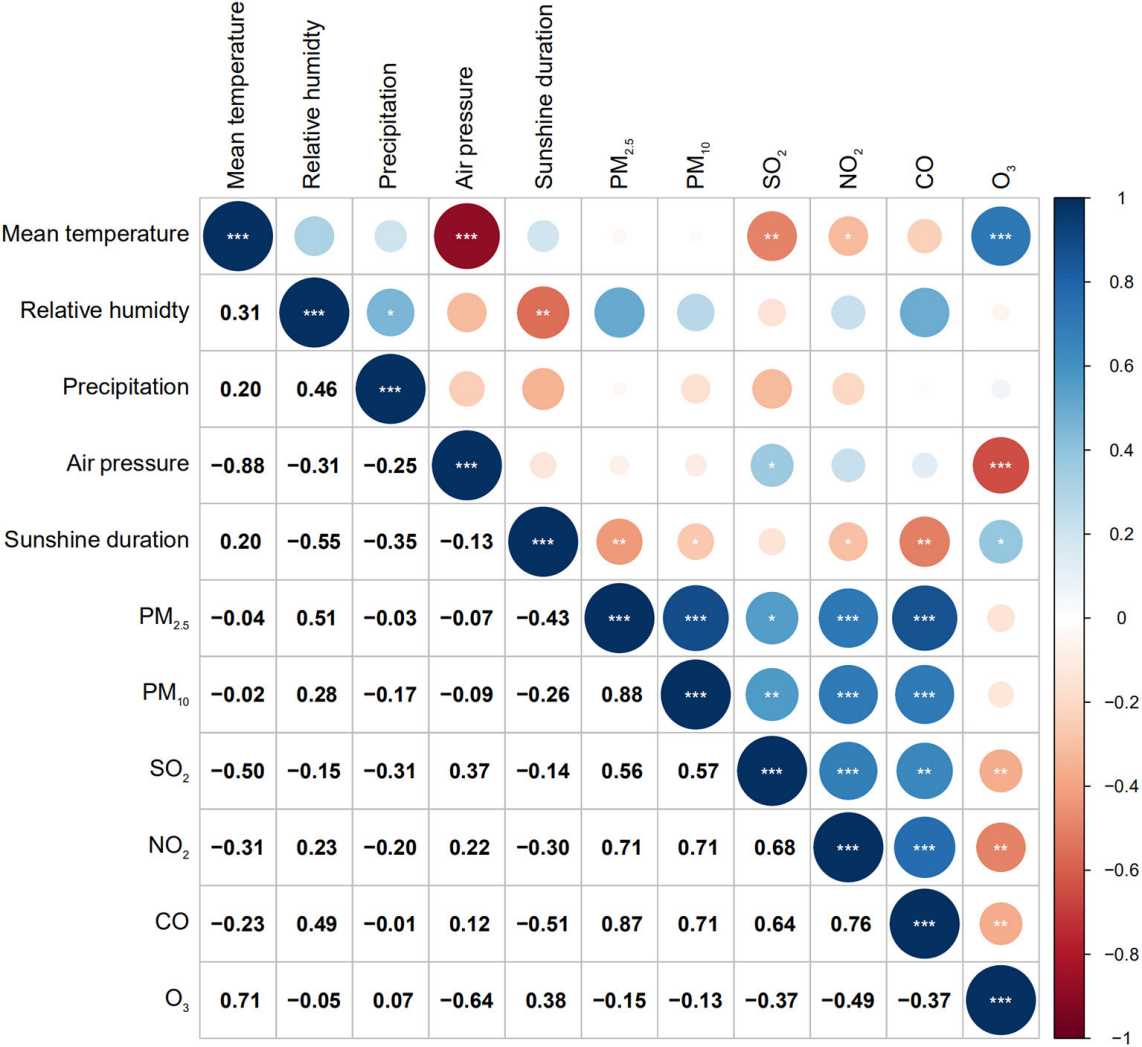
Spearman correlation coefficients between daily weather conditions and air pollutants in Beijing, China during 2013–2018. *P < 0.05; **P < 0.01; ***P < 0.001.

Considering the over-dispersion of daily AMI hospitalizations, we used a quasi-Poisson generalized linear model combined with a distributed lag model (DLM) to explore the impact of extreme precipitation on AMI hospitalizations (34). Figure 1 shows the correlation coefficients among daily weather variables and air pollution data using Spearman analysis. Mean temperature had a high negative correlation with barometric pressure (*r* = −0.88, P < 0.001), whereas correlations of PM_2.5_ with PM_10_ and CO were highly positive (*r* = 0.88, *P* < 0.001; *r* = 0.87, *P* < 0.001, respectively). The correlations between other variables were low. Hence, barometric pressure, PM_10_ and CO were not included in the final model. The potential confounding factors, including daily mean temperature, relative humidity, sunshine duration, PM_2.5_, NO_2_, SO_2_, O_3_, day of the week, official holidays and long-term trends were controlled in the model. The equation was described as follows:


(1) $$\begin{array}{l} Log\left[E\left(Y_{t}\right)\right]=\alpha +cb\left(EP_{t},\;lag,\;3\right)+ns\left(Temp_{t},\;3\right)+ns\left(RH_{t},\;3\right)\\ \;+ns\left(SD_{t},\;3\right)+ns\left(PM2.5,\;3\right)+ns\left(NO2,\;3\right)+ns\left(SO2,\;3\right)\\ \;+ns\left(O3,\;3\right)+ns\left(time_{t},\;6/per\;year\right)+\eta Holiday_{t}\\ \;+\gamma DOW_{t} \end{array}$$

where E (Y_t_) denoted the expected daily number of AMI hospitalizations on day t. α was the intercept. EP_t_ was an ordinal categorical variable labeled by 0 (no precipitation), 1 (moderate precipitation) and 2 (extreme precipitation). cb represented the cross-basis function, including a linear function and a natural cubic spline function with 3 df to assess the linear exposure-response relationship and non-linear lagged effects of extreme precipitation, respectively. ns () meant the natural cubic spline function. Temp_t_, RH_t_, and SD_t_ referred to mean temperature, relative humidity, and sunshine duration on day t with 3 dfs, respectively. We used an ns with 6 df per year to control the long-term trends and seasonality. Holiday_t_ was a binary variable, representing official holidays. DOW represented the day of the week on day t as a categorical variable. η, γ were the coefficients of the corresponding variables. According to the previous literature and lowest quasi-Akaike Information Criteria (Q-AIC), we fitted a lag period of up to 14 days in the models to capture the single and cumulative effects of extreme precipitation (17, 33). To identify the susceptible subpopulations for timely and effective public health interventions, we further performed subgroup analyses by gender (male and female) and age (20–64 years old and ≥65 years old).

We also calculated attributable fraction (AF) and attributable number (AN) to better reveal the burden of AMI associated with extreme precipitation using the forward perspective method (35). The following two formulas were applied:


AFt= (RRt-1)/RRt             ANt=AFt*Nt

where the RR was the largest cumulative relative risk (CRR) value calculated by extreme precipitation (variable value = 2) relative to no precipitation (variable value = 0). N_t_ represented the number of AMI cases on day t. All statistical analyses were performed with R software (version 4.1.0.). Statistical significance was considered as a two-sided p < 0.05.

### Sensitivity analysis

We performed several sensitivity analyses to test the robustness of our findings. We varied the df of mean temperature (3–5 df), relative humidity (3–5 df), sunshine duration (3–5 df), PM_2.5_ (3–5 df), SO_2_ (3–5 df), NO_2_ (3–5 df), O_3_ (3–5 df) and time (6-8 df) in the model. Furthermore, we used the other four dividing points (90th, 92.5th, 97.5th, and 99th percentiles) for defining extreme precipitation.

## Results

**Figure 2:**
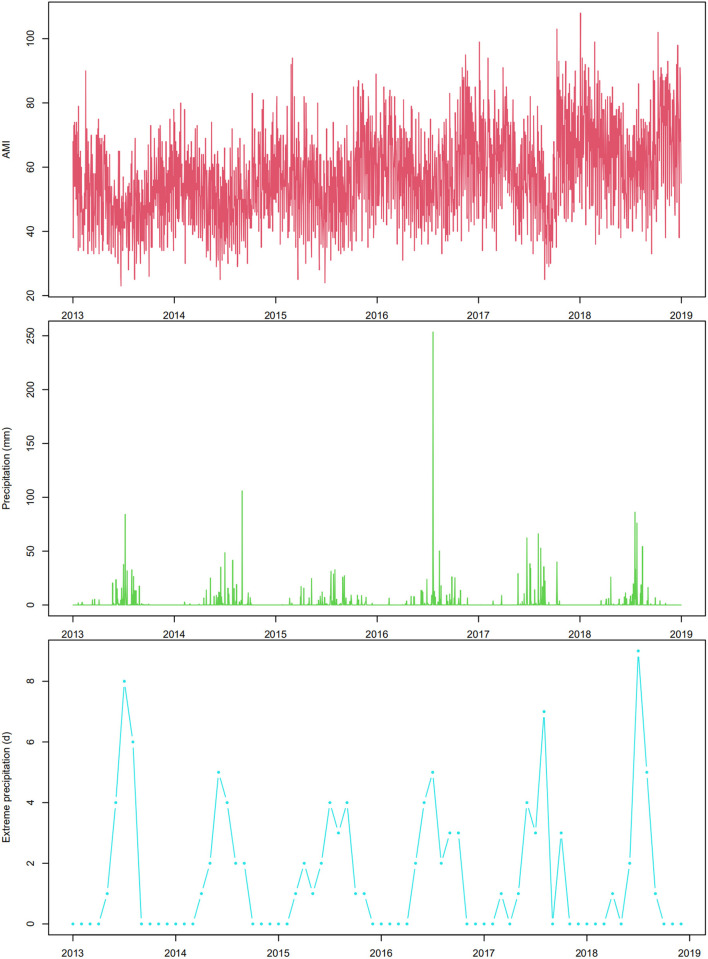
The time-series plot of daily AMI hospitalizations, daily precipitation, and monthly extreme precipitation events in Beijing, China during 2013–2018.

Table 1 shows the descriptive statistics of daily AMI hospitalizations, meteorological factors, and air pollutants in Beijing, China during 2013–2018. A total of 124,760 AMI hospitalizations among Beijing residents were recorded during the study period, with an average of 56.9 per day. There were much more cases in males (69.1%) than in females (30.9%). The percentage of patients aged ≥65 years old (53.7%) was higher than that of patients aged 20–64 years old (46.3%). The average values of daily precipitation, mean temperature, relative humidity, air pressure, and sunshine duration were 1.5 mm, 13.8°C, 52.1%, 1,012.9 hPa, and 6.7 h, respectively. The maximum daily precipitation was 146.6 mm. Figure 2 shows the distribution of daily AMI hospitalizations and precipitation in Beijing during the study period. A total of 110 days experienced extreme precipitation events (daily precipitation was ≥7.6 mm) during the 6 years. Most extreme precipitation days were in the warm seasons (May to October).

**Table 1:** Descriptive statistics of daily AMI hospitalizations, weather conditions, and air pollution in Beijing, China during 2013–2018.

**Variables**	**Number (%)**	**Mean (SD)**	**Minimum**	**Percentiles**			**Maximum**
				**25th**	**50th**	**75th**	
Total AMI cases	124,760 (100)	56.9 (13.6)	23	47	56	66	108
**Gender**							
Male	86,176 (69.1)	39.3 (10.0)	13	32	38	46	85
Female	38,584 (30.9)	17.6 (5.6)	3	14	17	21	41
**Age**							
20–64 years old	57,813 (46.3)	26.4 (6.8)	7	22	26	31	55
≥65 years old	66,947 (53.7)	30.6 (9.0)	8	24	30	37	68
**Meteorological variables**							
Rainfall (mm)	/	1.5 (8.3)	0.0	0.0	0.0	0.0	253.5
Mean temperature (°C)	/	13.8 (11.2)	−14.3	2.9	15.4	24.1	32.6
Relative humidity (%)	/	52.1 (20.0)	8.0	36.0	52.0	68.0	99.0
Barometric pressure (hPa)	/	1,012.9 (10.2)	990.0	1,004.2	1,013.0	1,021.1	1,040.0
Sunshine duration (h)	/	6.7 (4.0)	0.0	3.6	7.8	9.7	14.1
**Air pollutants**							
PM_2.5_ (μg/m^3^)	/	71.9 (63.3)	3.9	27.6	54.1	95.2	475.4
PM_10_ (μg/m^3^)	/	102.3 (73.9)	7.1	51.4	85.5	131.0	956.1
NO_2_ (μg/m^3^)	/	47.9 (23.3)	6.7	31.5	42.5	59.3	156.1
SO_2_ (μg/m^3^)	/	13.7 (16.9)	2.0	3.7	7.2	15.8	138.4
CO (mg/m^3^)	/	1.1 (0.9)	0.2	0.6	0.9	1.3	8.0
O_3_ (μg/m^3^)	/	68.7 (51.3)	2.2	32.4	58.3	91.0	331.1

AMI, acute myocardial infarction; SD, standard deviation; PM_2.5_, particulate matter with an aerodynamic diameter < 2.5 μm; PM_10_, particulate matter with an aerodynamic diameter < 10 μm; CO, carbon monoxide; SO_2_, sulfur dioxide; NO_2_, nitrogen dioxide; O_3_, ozone.

**Figure 3:**
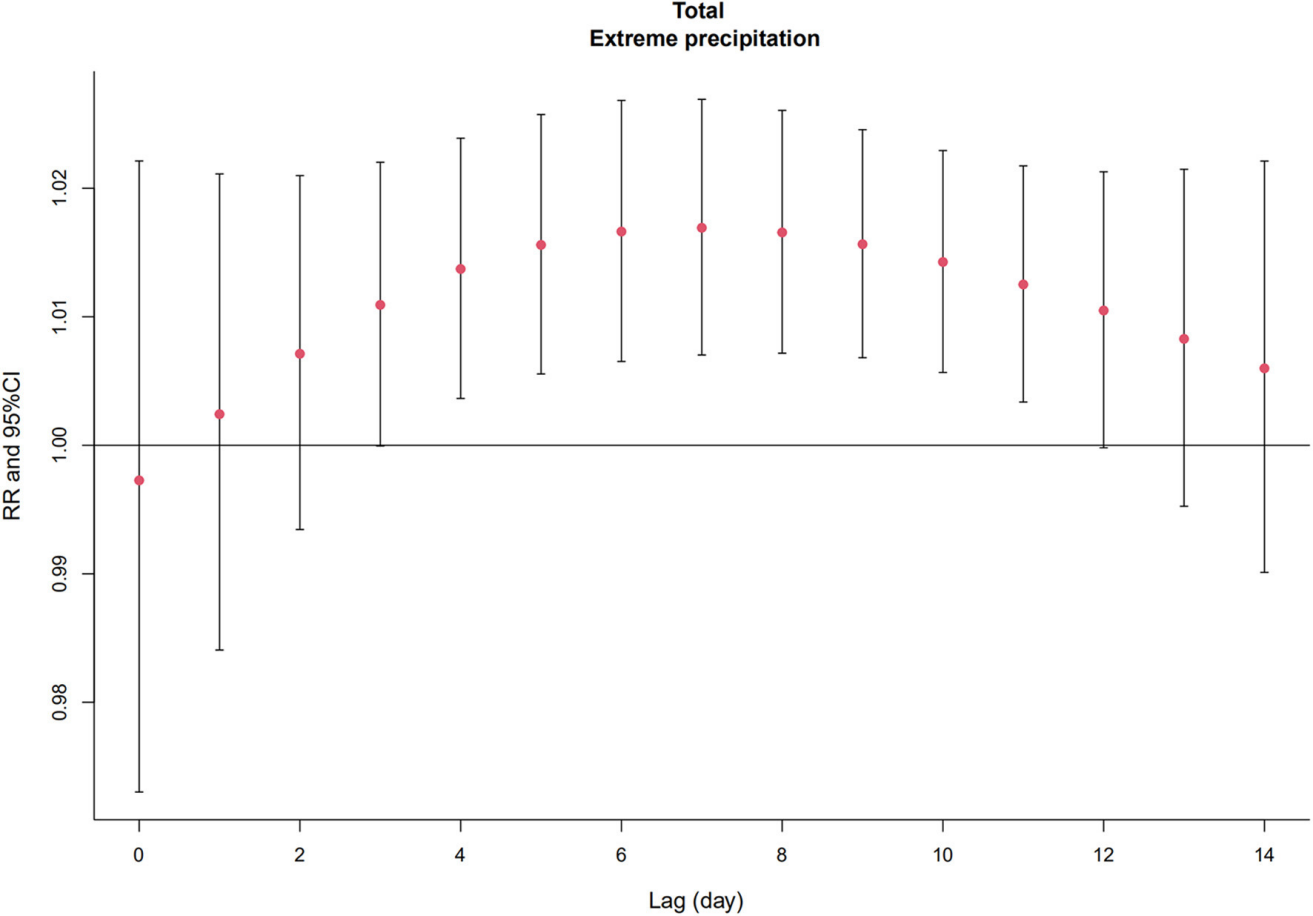
The relative risk (RR) and 95% confidence interval (CI) of extreme precipitation on total AMI hospitalizations on different lag days in Beijing, China from 2013 to 2018.

**Figure 4:**
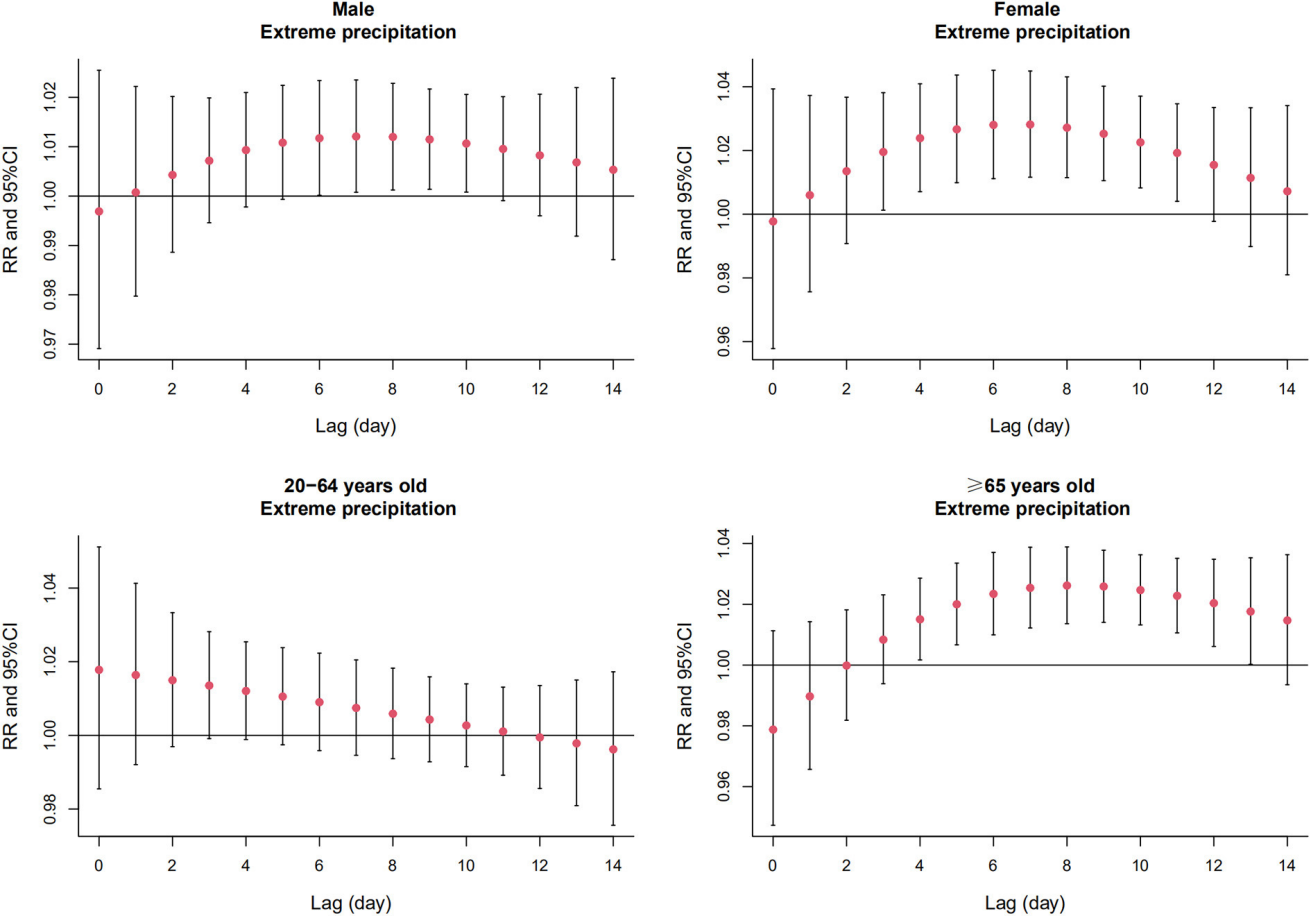
The relative risk (RR) and 95% confidence interval (CI) of extreme precipitation on AMI hospitalizations of subgroups on different lag days in Beijing, China from 2013 to 2018.

Figure 3 and Supplementary Table S1 depict the relative risk (RR) of AMI hospitalizations due to extreme precipitation in the total study population at different lag days. The significant effect of extreme precipitation on AMI hospitalizations began at Lag 4 [RR = 1.014, 95% confidence interval (CI): 1.004, 1.024] and continued to Lag 11 (RR = 1.013, 95% CI: 1.003, 1.022), with the largest single-day effect at Lag 7 (RR = 1.017, 95% CI: 1.007, 1.027). No significant association was found between AMI hospitalizations and extreme precipitation at other lag days. Figure 4 and Supplementary Table S1 reveal the results of the subgroup analysis stratified by gender and age. The lagged effect patterns of both males and females were similar to that of the total population, but the significant lagged effects of extreme precipitation lasted much longer in females (Lag 3-Lag 11) than those in males (Lag 6-Lag 10). For different age groups, people aged ≥ 65 years old were more susceptible to extreme precipitation. The significant association appeared from Lag 4 (RR = 1.015, 95% CI: 1.002, 1.029) to Lag 13 (RR = 1.018, 95% CI: 1.000, 1.035), with the largest effect at Lag 8 (RR = 1.026, 95% CI: 1.014, 1.039). However, no such association was detected in the people aged 20–64 years old.

Table 2 shows the CRR of AMI hospitalizations associated with extreme precipitation. Among the total population, the significant cumulative lag effects of extreme precipitation occurred at Lag 0–8 (CRR = 1.101, 95% CI: 1.007, 1.204), and persisted to Lag 0–14 with the largest effect (CRR = 1.177, 95% CI: 1.045, 1.326). In different subgroups, the significant cumulative effect of extreme precipitation on females appeared at Lag 0–7 (CRR = 1.152, 95% CI: 1.001, 1.326) and lasted to Lag 0–14 (RR = 1.308, 95% CI: 1.074, 1.593). For people aged ≥65 years, the significant CRR started from Lag 0–10 (CRR = 1.145, 95% CI: 1.006, 1.303) and persisted until Lag 0–14 (CRR = 1.234, 95% CI: 1.053, 1.445). Yet there was no statistical significance for males and people aged 20–64 years old.

**Table 2:** Cumulative relative risks (95% CI) of AMI hospitalizations associated with extreme precipitation among the total population and different subgroups in Beijing, China from 2013 to 2018.

**Lag days**	**Total**	**Male**	**Female**	**20–64 years old**	**≥65 years old**
0–0	0.997 (0.973, 1.022)	0.997 (0.969, 1.025)	0.998 (0.958, 1.039)	1.018 (0.985, 1.051)	0.979 (0.947, 1.011)
0–1	1.000 (0.958, 1.044)	0.998 (0.950, 1.048)	1.004 (0.935, 1.078)	1.034 (0.978, 1.094)	0.969 (0.915, 1.026)
0–2	1.007 (0.952, 1.065)	1.002 (0.940, 1.068)	1.017 (0.927, 1.116)	1.050 (0.976, 1.130)	0.969 (0.899, 1.043)
0–3	1.018 (0.954, 1.086)	1.009 (0.937, 1.087)	1.037 (0.931, 1.155)	1.064 (0.977, 1.159)	0.977 (0.896, 1.065)
0–4	1.032 (0.961, 1.108)	1.018 (0.938, 1.105)	1.062 (0.943, 1.195)	1.077 (0.981, 1.183)	0.991 (0.902, 1.090)
0–5	1.048 (0.971, 1.131)	1.030 (0.943, 1.124)	1.090 (0.961, 1.237)	1.088 (0.985, 1.203)	1.011 (0.914, 1.119)
0–6	1.065 (0.983, 1.155)	1.042 (0.949, 1.143)	1.121 (0.980, 1.281)	1.098 (0.988, 1.221)	1.035 (0.930, 1.152)
0–7	1.083 (0.995, 1.179)	1.054 (0.956, 1.162)	1.152 (1.001, 1.326)*	1.106 (0.990, 1.237)	1.061 (0.948, 1.188)
0–8	1.101 (1.007, 1.204)*	1.067 (0.963, 1.182)	1.183 (1.021, 1.372)*	1.113 (0.990, 1.251)	1.089 (0.967, 1.226)
0–9	1.118 (1.019, 1.228)*	1.079 (0.969, 1.201)	1.213 (1.040, 1.416)*	1.118 (0.989, 1.263)	1.117 (0.987, 1.265)
0–10	1.134 (1.029, 1.251)*	1.091 (0.975, 1.220)	1.241 (1.056, 1.458)*	1.121 (0.986, 1.274)	1.145 (1.006, 1.303)*
0–11	1.149 (1.037, 1.272)*	1.101 (0.979, 1.238)	1.265 (1.069, 1.496)*	1.122 (0.981, 1.282)	1.171 (1.023, 1.340)*
0–12	1.161 (1.043, 1.291)*	1.110 (0.982, 1.255)	1.284 (1.077, 1.531)*	1.121 (0.975, 1.290)	1.195 (1.037, 1.376)*
0–13	1.170 (1.046, 1.309)*	1.118 (0.982, 1.271)	1.299 (1.079, 1.563)*	1.119 (0.965, 1.296)	1.216 (1.048, 1.410)*
0–14	1.177 (1.045, 1.326)*	1.124 (0.979, 1.289)	1.308 (1.074, 1.593)*	1.114 (0.953, 1.304)	1.234 (1.053, 1.445)*

CI, confidence interval; AMI, acute myocardial infarction.

* p < 0.05.

Because the CRR of extreme precipitation on AMI hospitalizations increased until the 14th day, we selected the CRR value of Lag 0–14 to calculate attributable fractions (AF) and attributable numbers (AN). Table 3 shows the AF and AN of AMI hospitalizations due to extreme precipitation. Total AF was 0.68% (95% CI: 0.20%, 1.12%) and a total of 854 (95% CI: 244, 1,395). AMI hospitalizations were attributed to extreme precipitation from 2013 to 2018. As for the subgroup analysis, extreme precipitation caused a higher disease burden in females than in males. The AF and AN of females were 1.06% (95% CI: 0.70%, 1.67%) and 409 (95% CI: 271, 646), respectively; whereas those of the males were 0.50% (95% CI: −0.10%, 1.02%) and 435 (95% CI: −85, 883), respectively. For different age subgroups, the attributable AMI burdens owing to extreme precipitation in people aged ≥65 years old (AF = 0.82%, 95% CI: 0.22%, 1.34%; AN = 551, 95% CI: 146, 895) were higher than those in people aged 20–64 years old (AF = 0.49%, 95% CI: −0.24%, 1.12%; AN = 283, 95% CI: −137, 646).

**Table 3:** Attributable fractions (%) and attributable number (95% CI) of AMI hospitalizations due to extreme precipitation in Beijing, China from 2013 to 2018.

	**AF**	**AN**
Total	0.68% (0.20%, 1.12%)	854 (244, 1395)
Male	0.50% (−0.10%, 1.02%)	435 (−85, 883)
Female	1.06% (0.70%, 1.67%)	409 (271, 646)
20–64 years old	0.49% (−0.24%, 1.12%)	283 (−137, 646)
≥65 years old	0.82% (0.22%, 1.34%)	551 (146, 895)

CI, confidence interval; AMI, acute myocardial infarction; AF, attributable fraction; AN, attributable number.

The sensitivity analyses indicated that the results were still robust after changing the df for cofounding variables: mean temperature (3–5 df), relative humidity (3–5 df), sunshine duration (3–5 df), PM_2.5_ (3–5 df), SO_2_ (3–5 df), NO_2_ (3–5 df), O_3_ (3–5 df) and time (6–8 df) in the model (see Supplementary materials; Supplementary Figures S1–S5). Also, applying the other four cut-off values (90th, 92.5th, 97.5th, and 99th percentiles) to define extreme precipitation did not change the results substantially (Supplementary Figure S6).

## Discussion

We found that extreme precipitation was significantly correlated with a higher risk of AMI hospitalizations from 2013 to 2018 in Beijing, China. The significant single-day lag effect lasted from Lag 4 to Lag 11 and the cumulative lag effect continued to Lag 0–14. The AF and AN of AMI hospitalizations from extreme precipitation were 0.68% and 854, respectively. Females and older people (≥65 years old) were more vulnerable to extreme precipitation than males and the younger (20–64 years old). With more frequent and intense extreme weather events as global climate change, our findings may have important implications on our understanding of the adverse health impact of extreme precipitation, and provide evidence for developing practical measures and public health recommendations to prevent AMI incidence in at least 2 weeks after extreme precipitation events.

Some previous studies have supported our findings, reporting positive associations between precipitation and AMI, whereas no study has investigated the impact of extreme precipitation on AMI (18–20). An early study showed that mortality of IHD was positively correlated with rainfall in England and Wales. However, the association was reported only by the correlation coefficient (r) and the corresponding *p*-value based on yearly data rather than daily data (18). Ebi and colleagues found that per 0.5 mm increase of precipitation was significantly associated with an elevated percentage of AMI hospital admissions in people aged ≥ 70 in Los Angeles, whereas no significant association was detected in San Francisco and Sacramento (19). Another study conducted in Switzerland reported that per 1 mm increment of rainfall correlated with more hospitalizations for AMI in the Alps and their adjacent regions, whereas no statistically significant impact was shown in other areas (20). On the contrary, a nationwide study in Sweden suggested a significant negative relationship between rain precipitation and the daily incidence rate of AMI (5). A retrospective analysis in Italy demonstrated that the rates of primary percutaneous coronary angioplasty for ST-segment elevation myocardial infarction (STEMI) were increased by lower rainfall only in winter (22). Another study in Japan also suggested that lower precipitation was independently associated with STEMI occurrence (25). However, some other studies found no significant association (6, 21, 23, 24). The inconsistent results across the studies may be due to various weather conditions, characteristics of study populations, and statistical methods. More multi-center studies from different regions should be encouraged.

The biological mechanisms between extreme precipitation and AMI onset remain unclear with the following potential explanations. First, extreme weather events related to climate change have been proven to exert adverse impacts on mental health (32, 36). Patients with mental disorders tend to have higher risks of both AMI and stroke (37). Second, extreme precipitation events may increase the humidity in the environment. It is hypothesized that the ability of perspiration and body temperature homeostasis could be affected in a high humidity environment, leading to respiratory fatigue and higher heart rate (38), which increase myocardial oxygen consumption. Third, extreme precipitation may help the transmission of viruses and bacteria, resulting in increased risks of influenza and other acute respiratory infectious diseases (39, 40). Mounting evidence has shown that AMI can be triggered by acute infection (41). Future studies should unravel more specific mechanisms.

Our subgroup analysis showed that females had a higher burden of AMI hospitalizations caused by extreme precipitation than males. One possible explanation is that females seem to be more sensitive to environmental stress and mental illness than males (42). As for the age subgroups, the elderly (≥65 years old) seemed more susceptible to the impact of extreme precipitation than younger people aged 20–64 years old, which is consistent with a prior study focusing on ischemic stroke (17). The reason may point to lower adaption capacity to environmental changes, higher prevalence of basic diseases and declining immune immunity of the elderly population.

The study has several strengths. Firstly, to our knowledge, this is the first study to assess the association between extreme precipitation and AMI hospitalizations. Secondly, the database used in this study covers almost all the AMI hospitalizations in Beijing during the 6 years. Complete data with a large sample size (over 124,000 hospitalizations) enhance the reliability of our results. Thirdly, we further explored the lagged effects using DLM, identified vulnerable populations by subgroup analysis, and calculated the attributable burden, which provided comprehensive information for the targeted prevention measures for AMI in Beijing.

Nevertheless, some major limitations should be noticed. Firstly, we collected weather and air pollution data from fixed monitoring sites to roughly represent individual exposures of the whole study population. Many potential confounders, such as medical history, smoking status, and other individual-level factors were not included in the analysis for the lack of information. Therefore, the ecological fallacy is a concern and the findings should be further confirmed in large-scale individual-based studies. Secondly, because the distribution of precipitation values during the study period was completely heterogeneous with too many days equal to 0, the precipitation was classified into a categorical variable in the present study. A more detailed exposure-response relationship between AMI and precipitation as a continuous variable should be explored in the future. Furthermore, the interaction between extreme precipitation and other environmental variables is needed to reveal in more sophisticated models. Thirdly, the results from a single city limited their generalization to other regions with different weather or socio-economic conditions. Future studies are warranted to assess the influence of extreme precipitation on AMI occurrence to confirm and extend our research findings.

The study suggested that extreme precipitation was associated with a higher risk of AMI hospitalizations in Beijing, China from 2013 to 2018. People aged ≥ 65 years and females are potentially vulnerable populations to extreme precipitation-related AMI hospitalizations. Our results may arouse more attention to AMI associated with extreme precipitation, especially for the elderly and females, and provide evidence for implementing policies and measures against extreme precipitation in the context of climate change.

## Data availability statement

The original contributions presented in the study are included in the article/Supplementary material, further inquiries can be directed to the corresponding author.

## Author contributions

YC, ZC, and ZF contributed to the conception and design of the study. YC, JF, DK, and XL organized the database. ZC, YaL, and YH performed the statistical analysis. YC and ZC wrote the first draft of the manuscript. YZ, YiL, and ST wrote sections of the manuscript. All authors contributed to manuscript revision, read, and approved the submitted version.

## Funding

This work was supported by National Natural Science Foundation of China (Grant Numbers 12126602, 41450006, 91643208); Chinese Academy of Medical Sciences (CAMS). Initiative for Innovative Medicine (Grant Number 2017-I2M-2-001); and National Environmental Protection Non-Profit Project (Grant Number 201509062).

## Conflict of interest

The authors declare that the research was conducted in the absence of any commercial or financial relationships that could be construed as a potential conflict of interest.

## Publisher's note

All claims expressed in this article are solely those of the authors and do not necessarily represent those of their affiliated organizations, or those of the publisher, the editors and the reviewers. Any product that may be evaluated in this article, or claim that may be made by its manufacturer, is not guaranteed or endorsed by the publisher.
