# Supplementary material for: Association of extreme precipitation with hospitalizations for acute myocardial infarction in Beijing, China: A time-series study
Source: Front Public Health. 2022 Sep 27;10:1024816. doi: 10.3389/fpubh.2022.1024816 (PMC9551252; doi:10.3389/fpubh.2022.1024816)
Supplement: Supplementary file 1 [file Table_1.DOCX]

Supplementary Material

# Supplementary Figures and Tables

## Supplementary Figures


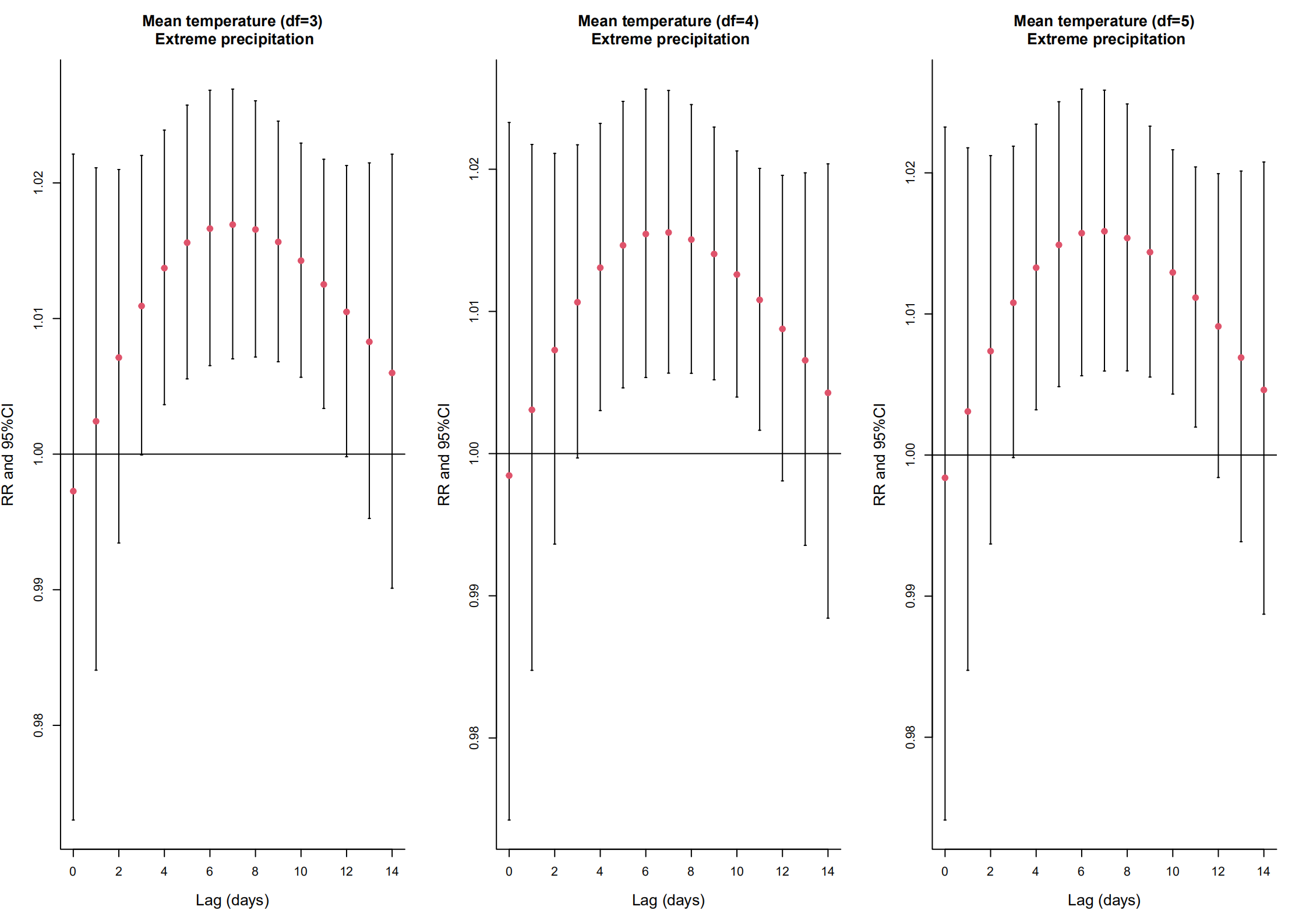


**Figure S1.** The lag effects of extreme precipitation on total AMI hospitalizations in the sensitivity analyses about changing df for mean temperature (3-5)


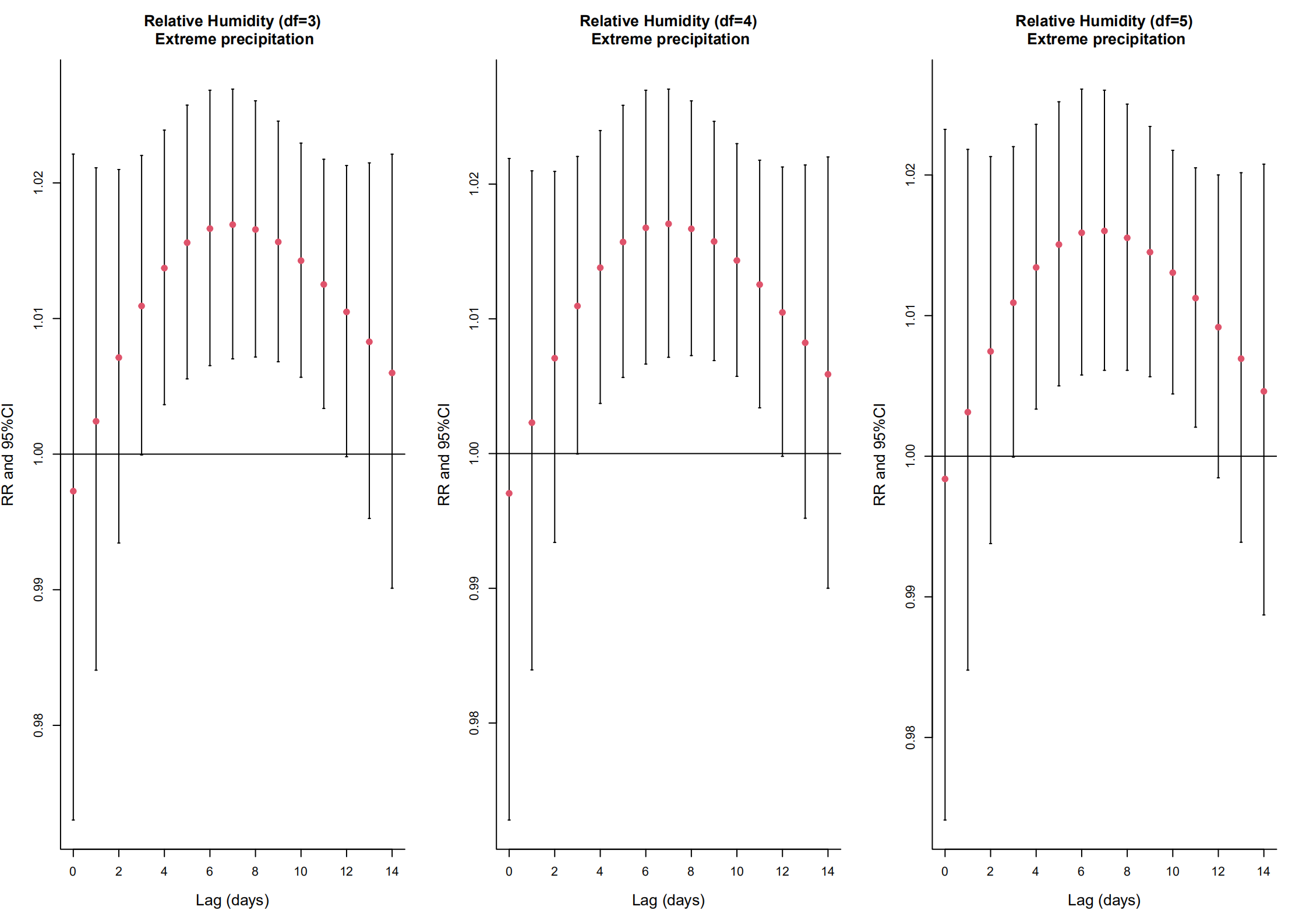


**Figure S2.** The lag effects of extreme precipitation on total AMI hospitalizations in the sensitivity analyses about changing df for relative humidity (3-5)


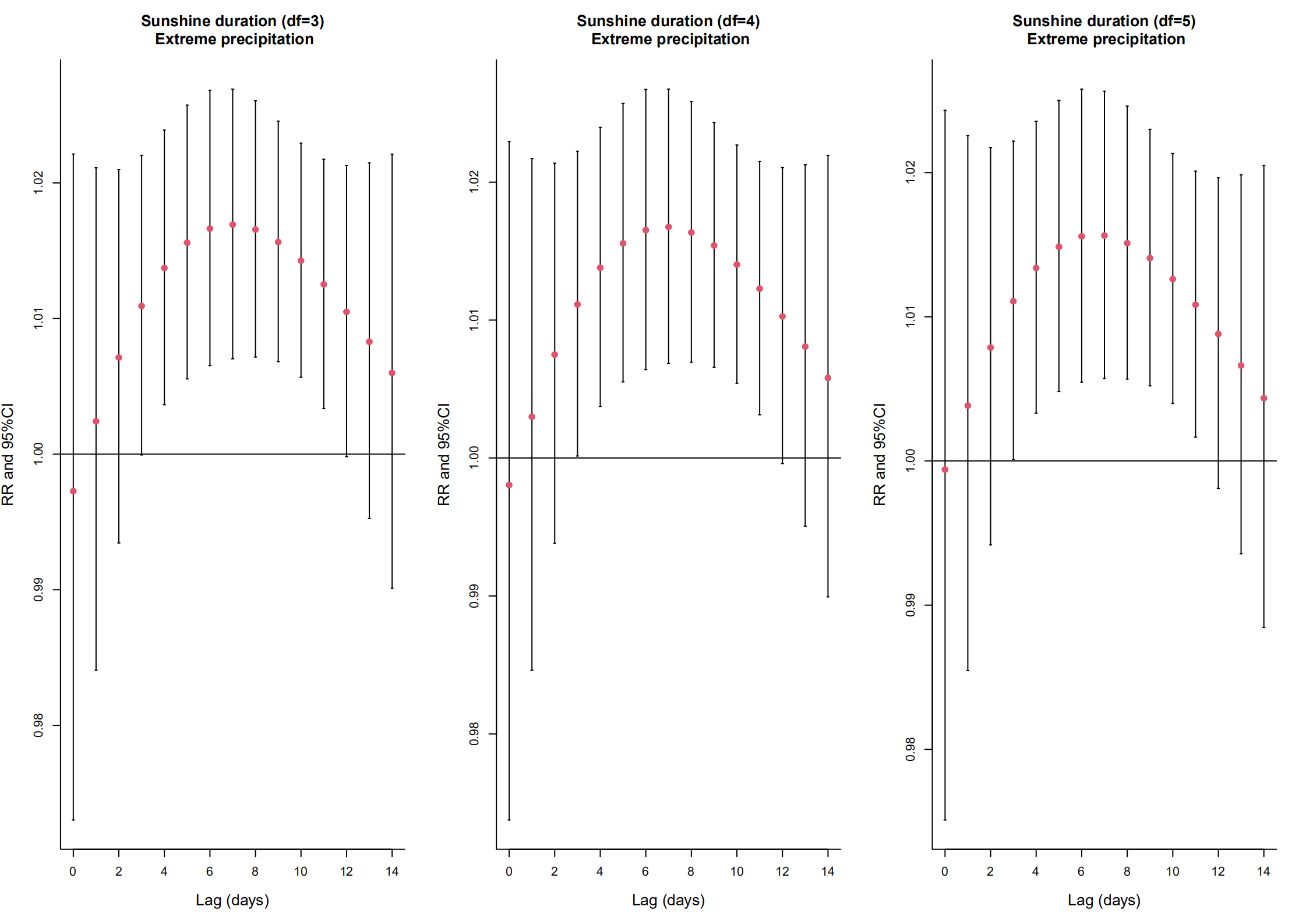


**Figure S3.** The lag effects of extreme precipitation on total AMI hospitalizations in the sensitivity analyses about changing df for sunshine duration (3-5)


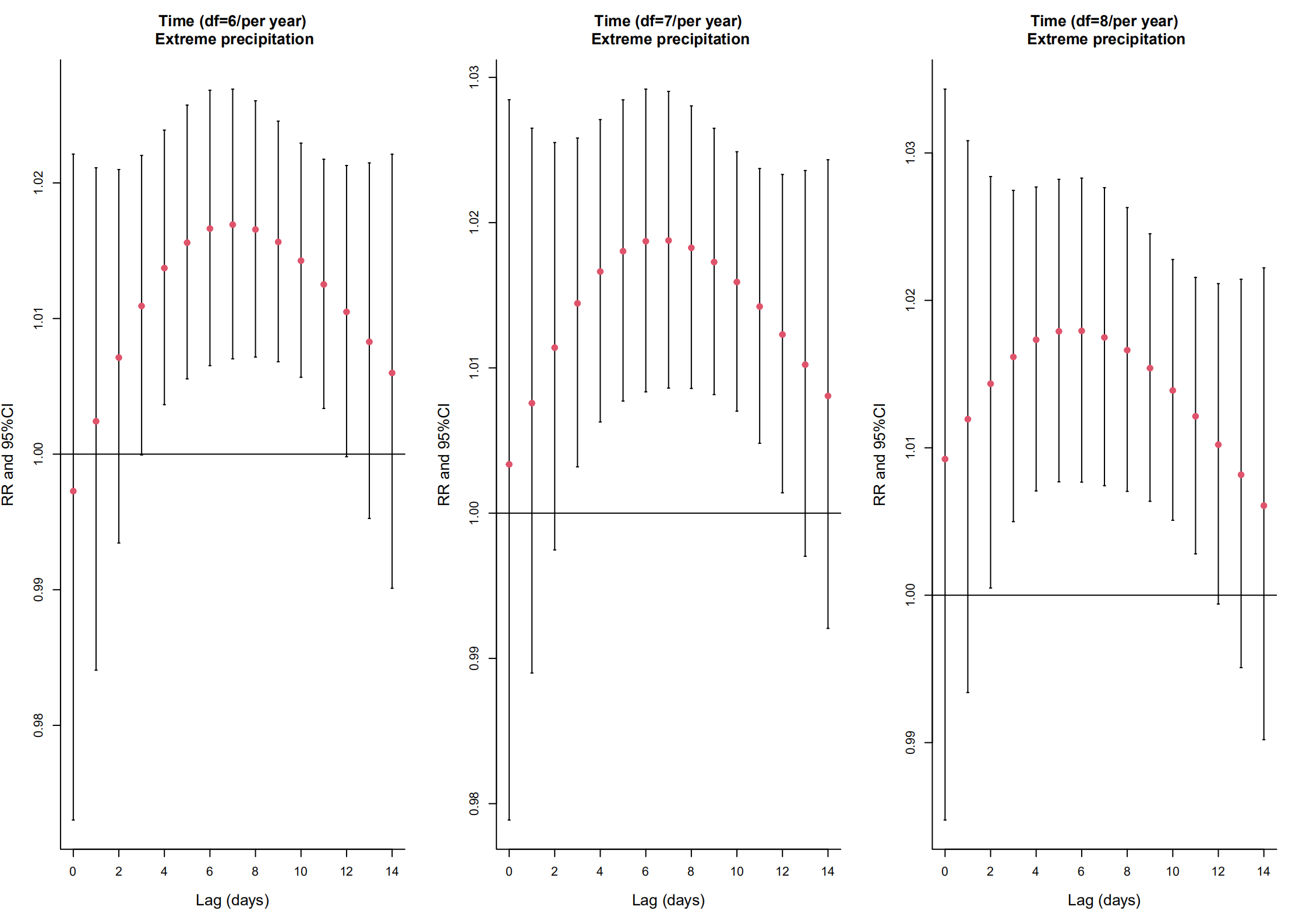


**Figure S4.** The lag effects of extreme precipitation on total AMI hospitalizations in the sensitivity analyses about changing df for time (6-8)


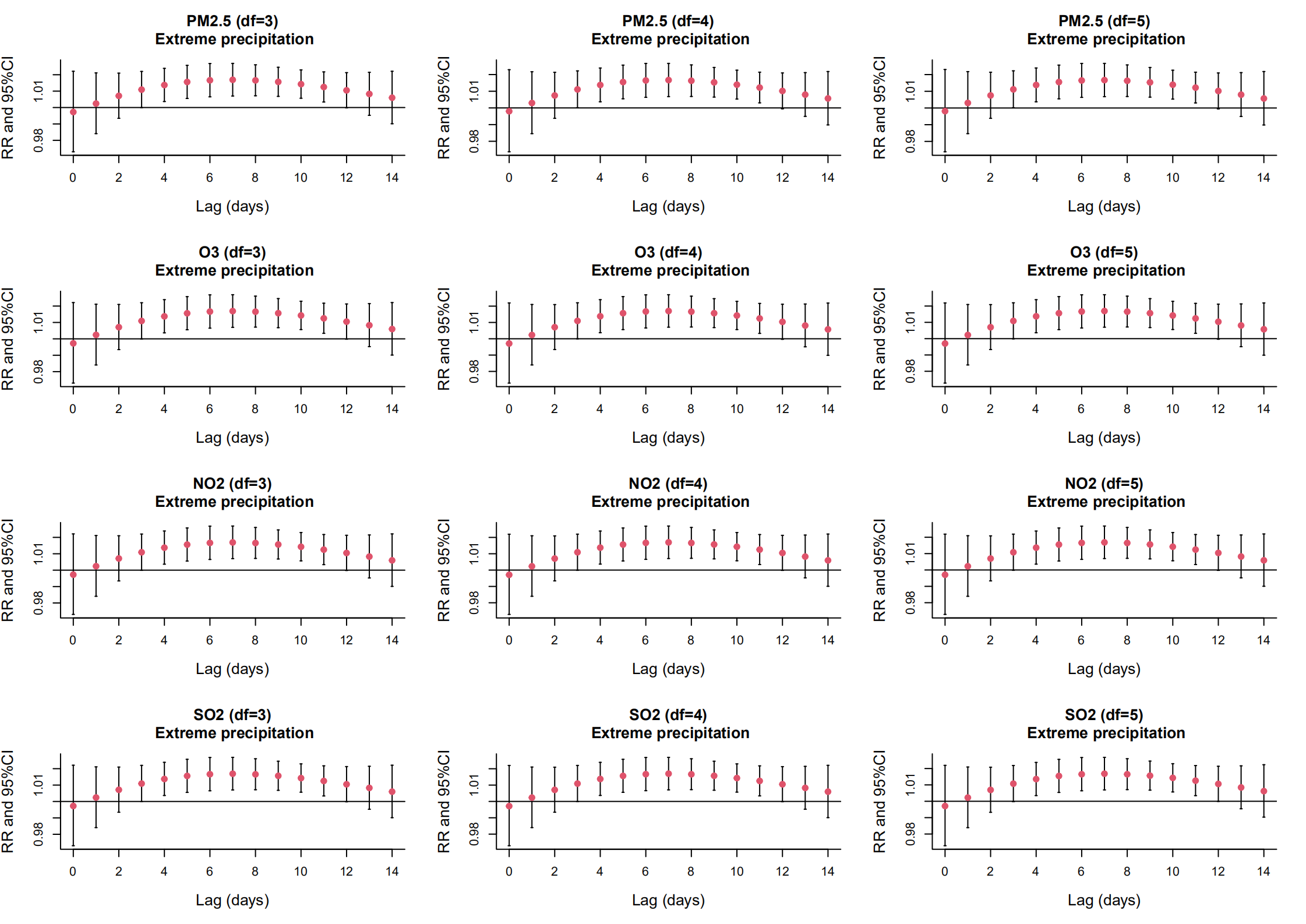


**Figure S5.** The lag effects of extreme precipitation on total AMI hospitalizations in the sensitivity analyses about changing df for air pollutants (3-5)


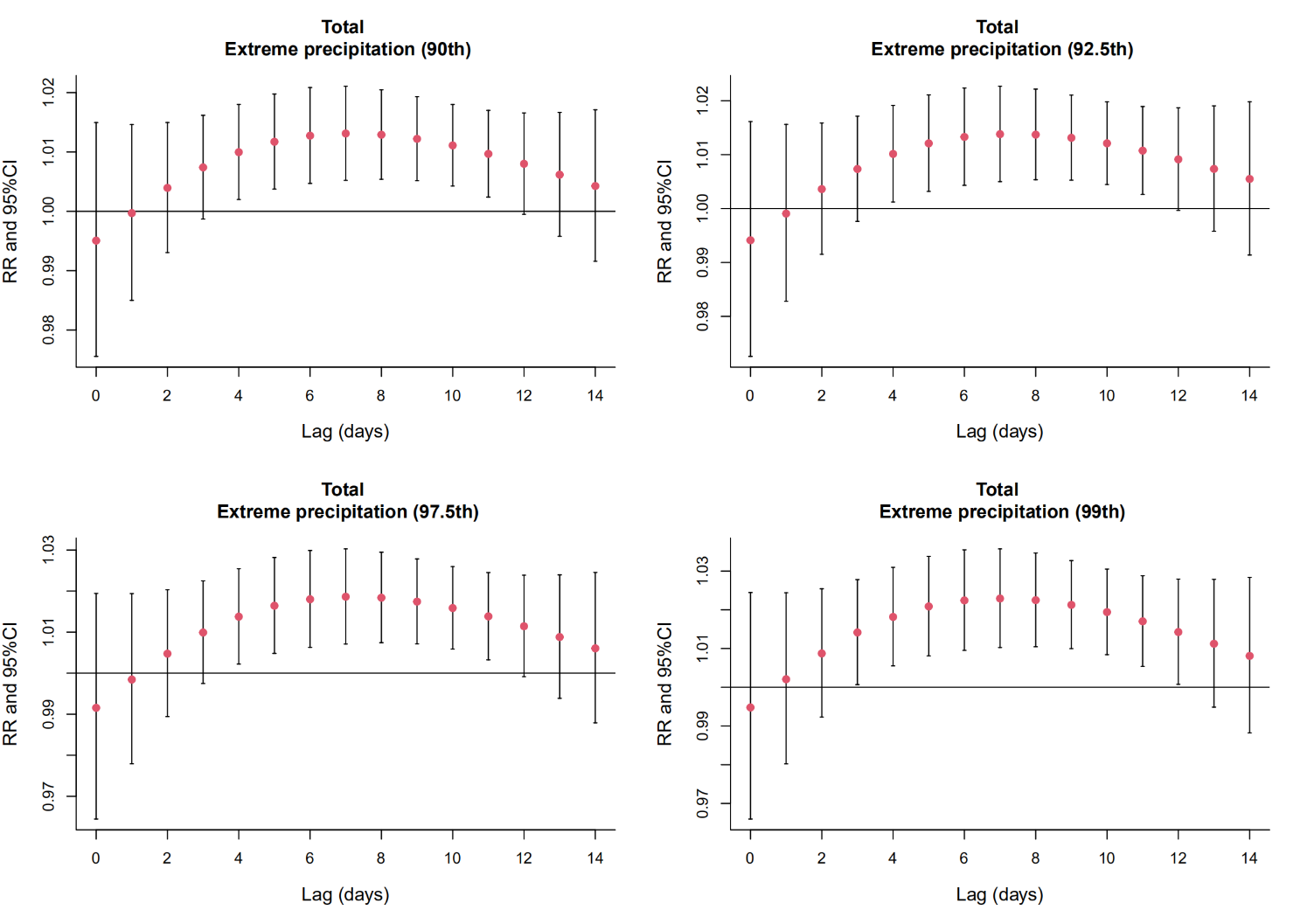


**Figure S6.** The lag effects of extreme precipitation on total AMI hospitalizations in the sensitivity analyses about using four different cut-off values (90th,92.5th, 97.5th and 99th percentile).

## Supplementary Tables

**Table S1** Single-day lag effects (RR with 95% CI) of extreme precipitation on AMI hospitalizations in the total population and different subgroups in Beijing, China from 2013 to 2018.

| Single-day lag | Total | Male | Female | 20-64 years old | ≥65 years old |
| --- | --- | --- | --- | --- | --- |
| 0 | 0.997 (0.973, 1.022) | 0.997 (0.969, 1.025) | 0.998 (0.958, 1.039) | 1.018 (0.985, 1.051) | 0.979 (0.947, 1.011) |
| 1 | 1.002 (0.984, 1.021) | 1.001 (0.980, 1.022) | 1.006 (0.976, 1.037) | 1.016 (0.992, 1.041) | 0.990 (0.966, 1.014) |
| 2 | 1.007 (0.993, 1.021) | 1.004 (0.989, 1.020) | 1.013 (0.991, 1.037) | 1.015 (0.997, 1.033) | 1.000 (0.982, 1.018) |
| 3 | 1.011 (1.000, 1.022) | 1.007 (0.995, 1.020) | 1.020 (1.001, 1.038)* | 1.014 (0.999, 1.028) | 1.008 (0.994, 1.023) |
| 4 | 1.014 (1.004, 1.024)* | 1.009 (0.998, 1.021) | 1.024 (1.007, 1.041)* | 1.012 (0.999, 1.025) | 1.015 (1.002, 1.029)* |
| 5 | 1.016 (1.006, 1.026)* | 1.011 (0.999, 1.022) | 1.027 (1.010, 1.044)* | 1.011 (0.997, 1.024) | 1.020 (1.007, 1.034)* |
| 6 | 1.017 (1.007, 1.027)* | 1.012 (1.000, 1.023)* | 1.028 (1.011, 1.045)* | 1.009 (0.996, 1.022) | 1.023 (1.010, 1.037)* |
| 7 | 1.017 (1.007, 1.027)* | 1.012 (1.001, 1.024)* | 1.028 (1.012, 1.045)* | 1.007 (0.995, 1.020) | 1.025 (1.012, 1.039)* |
| 8 | 1.017 (1.007, 1.026)* | 1.012 (1.001, 1.023)* | 1.027 (1.011, 1.043)* | 1.006 (0.994, 1.018) | 1.026 (1.014, 1.039)* |
| 9 | 1.016 (1.007, 1.025)* | 1.011 (1.001, 1.022)* | 1.025 (1.011, 1.040)* | 1.004 (0.993, 1.016) | 1.026 (1.014, 1.038)* |
| 10 | 1.014 (1.006, 1.023)* | 1.011 (1.001, 1.021)* | 1.023 (1.008, 1.037)* | 1.003 (0.992, 1.014) | 1.025 (1.013, 1.036)* |
| 11 | 1.013 (1.003, 1.022)* | 1.010 (0.999, 1.020) | 1.019 (1.004, 1.035)* | 1.001 (0.989, 1.013) | 1.023 (1.011, 1.035)* |
| 12 | 1.010 (1.000, 1.021) | 1.008 (0.996, 1.021) | 1.015 (0.998, 1.033) | 0.999 (0.986, 1.014) | 1.020 (1.006, 1.035)* |
| 13 | 1.008 (0.995, 1.021) | 1.007 (0.992, 1.022) | 1.011 (0.990, 1.033) | 0.998 (0.981, 1.015) | 1.018 (1.000, 1.035)* |
| 14 | 1.006 (0.990, 1.022) | 1.005 (0.987, 1.024) | 1.007 (0.981, 1.034) | 0.996 (0.976, 1.017) | 1.015 (0.994, 1.036) |

RR, relative risk; CI, confidence interval; AMI, acute myocardial infarction

*p<0.05
